# Psychiatric characteristics of pregnant crack users admitted to a referral center in Southern Brazil

**DOI:** 10.1192/j.eurpsy.2025.999

**Published:** 2025-08-26

**Authors:** M. B. Terra, J. V. E. Santos, N. A. F. da Silva, L. M. A. Sartes, J. B. Schuch, F. H. P. Kessler

**Affiliations:** 1 Department of Internal Medicine, Federal University of Health Sciences of Porto Alegre, Porto Alegre; 2 Department of Psychology, Federal University of Juiz de Fora, Juiz de Fora; 3 Department of Psychiatry and Legal Medicine, Federal University of Rio Grande do Sul - Graduate Program in Psychiatry and Behavioral Sciences, Porto Alegre, Brazil

## Abstract

**Introduction:**

Psychoactive substance use among pregnant women has reached alarmingly high rates and is considered a public health problem. Pregnancy is a period in which women become more sensitive and concerned about their well-being, in view of how that will affect their baby. Therefore, pregnancy becomes a favorable period for therapeutic intervention, especially with regard to drug use. Despite this, there is still a small number of studies that address the issue of female drug users in Brazil, especially due to stigma and prejudice.

**Objectives:**

Our main aim was to characterize the clinical and psychiatric profile of pregnant crack users in Brazil, with a focus on comorbidities, the severity of crack use, and the use of other substances.

**Methods:**

This was a cross-sectional study of 24 pregnant crack users admitted to a referral hospital for psychiatric disorders in pregnant women, in Porto Alegre, Brazil, over three years. Most women tend to remain hospitalized for a long time, often months, until giving birth. This scenario directly influenced the sample size of this study. The following instruments were applied: a clinical-obstetric questionnaire; the condensed version of the Addiction Severity Index; a diagnostic interview for psychoactive substance use based on DSM-5; the Mini International Neuropsychiatric Interview for DSM-IV; and the Semi-Structured Clinical Interview for DSM-IV Axis II Personality Disorders (SCID-II).

**Results:**

Most patients had severe crack dependence and used other substances, such as tobacco, cannabis, and alcohol. The median duration of crack use was three years, ranging between three and 12 years. Most women subsisted from illegal or informal activities; a fifth had previously been arrested and often had relationship problems. Twenty percent had HIV (n = 5), and 37.5% (n = 9) had syphilis. Borderline personality disorder was the most prevalent mental condition (62.5%), followed by suicidal tendencies (45.8%), hypomanic episodes due to substance use (37.5%), and past major depressive episodes (33.3%).

**Conclusions:**

This is one of the few studies exploring and characterizing social, economic and health aspects of pregnant crack users in Brazil. An alarmingly high prevalence of consumption of other drugs, psychiatric disorders, and difficult-to-treat personality disorders was observed in our study. Investigating the psychiatric profile of women who use substances is essential to minimize the impacts on the mother and child, optimize therapeutic approaches to comorbidities, and enable more effective relapse prevention.

**Disclosure of Interest:**

None Declared

